# A theoretical study on evaluating brain tumor changes in tumor treating fields therapy by impedance detection

**DOI:** 10.3389/fonc.2024.1443406

**Published:** 2024-09-04

**Authors:** Xing Li, Kaida Liu, Haohan Fang, Zirong Liu, Wei Gao, Ping Dai

**Affiliations:** ^1^ College of Automation Engineering, Nanjing University of Aeronautics and Astronautics, Nan Jing, Jiang Su, China; ^2^ Department of Radiotherapy, Shanghai Fourth People’s Hospital, School of Medicine, Tongji University, Shanghai, China

**Keywords:** tumor treating fields, tumor changes, brain tumor, impedance detection, evaluating effectiveness

## Abstract

TTFields is a novel FDA-approved technology utilized for treating glioblastoma multiforme (GBM) within the brain. Presently, the effectiveness of therapy is evaluated through MRI imaging at random two-month intervals. Electrical impedance is an important and effective parameter for reflecting changes in tissue properties. In TTFields treatment for brain tumors, electrodes attached to the scalp deliver electric field energy to the tumor region. We hypothesize that these electrodes can also serve as sensors to detect impedance changes caused by tumor alterations in real time, thus continuously assessing the effectiveness of the treatment. In this work, we propose and scrutinize this hypothesis by conducting an in silico study to confirm the potential feasibility of the proposed concept. Our results indicate that the impedance amplitude change measured between opposing TTFields electrode arrays utilizing voltage and frequency of 50 V and 200 kHz (typical TTFields treatment parameters), has enough resolution (> 1mm) and Signal-to-Noise Ratio (> 40 dB) to evaluate tumor size change in the head. The impedance detection technique may be a significant augmentation to TTFields cancer treatment, enabling the continuous evaluation of safety and efficacy throughout the procedure.

## 1 Introduction

Tumor Treating Fields (TTFields) constitute a safe and non-invasive technology for ablating malignant tissues. It relies on intermediate-frequency electric fields (100 kHz-500 kHz) of low intensity (< 3 V/cm) to impede the proliferation of cancer cells. This innovative technology was pioneered by Yoram Palti’s team in the early 2000s (1). Clinical evidence demonstrating the effectiveness of TTFields in prolonging the survival of GBM patients, importantly, without notable side effects. Consequently, the Food and Drug Administration (FDA) approved its use in GBM treatment (2, 3).

TTFields are delivered to the tumor by insulated electrode arrays that are directly applied to the patient’s shaved scalp (4). In the GBM treatment, TTFields are activated by the patient through controlling the portable power generator in the backpack. To achieve maximum therapeutic effect, a primary magnetic resonance image (MRI) should be done to confirm the exact position of tumor in the brain, and then treatment electrodes will be personalized attached on each patient. For this purpose, the NovoTal System (NovoTal, USA) offers commercial software designed to optimize electrode placements. A comprehensive description of the methodology for optimizing electrode placements and selecting treatment parameters can be found in (4).

The TTFields treatment differs significantly from other clinical tissue ablation techniques based on biophysical phenomena. Most traditional ablation techniques, for instance, microwave ablation occurs during a brief, acute surgical procedure. Surgeons or radiologists administer the ablative energy, guided by real-time medical imaging, and the procedure’s result can be evaluated shortly after its conclusion, typically through medical imaging assessments. In contrast, TTFields tissue ablation is an extended process that exclusively impacts replicating cells and involves the continuous delivery of electric fields over many months, and sometimes even years (5). As previously mentioned, the precise positioning of the electric field delivery electrodes is determined independently from the treatment itself. The electric fields are applied to the tumor typically for up to 18 hours each day (6). Due to the protracted nature of the TTFields ablation procedure, spanning months, it becomes challenging to continually assess its effectiveness throughout the treatment. Currently, the treatment’s efficacy is assessed through follow-up MRI scans, typically conducted at intervals of every two months (4, 7). This lack of continuous monitoring, compounded by the extended treatment duration, represents a limitation in the GBM treatment by TTFields.

**Figure 1:**
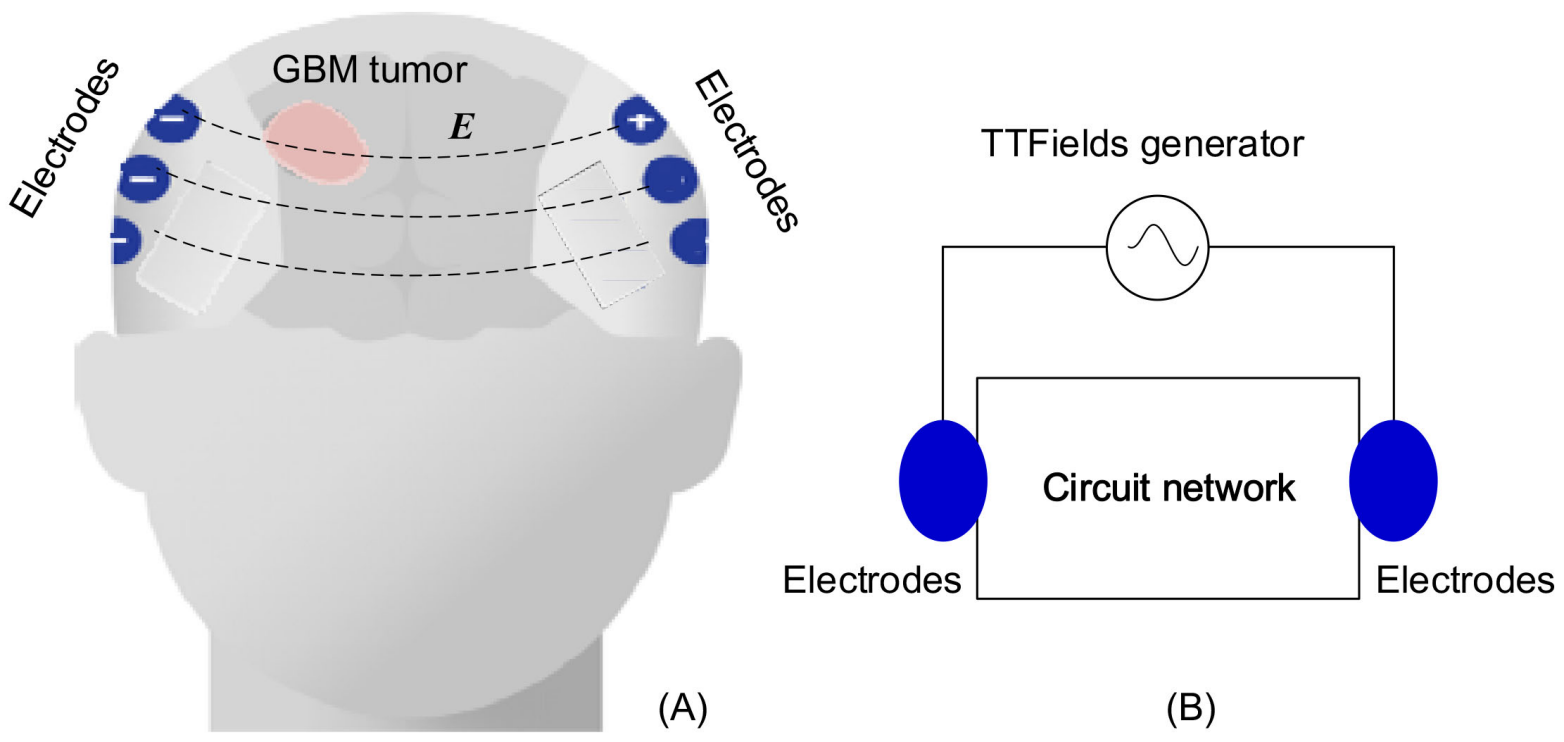
TTFields treatment on GBM: **(A)** configuration of electrode array, **(B)** equivalent lump model circuit.

TTFields electrodes are strategically positioned at predetermined locations, carefully calculated to optimize the delivery of electric fields to the specific location and size of the tumor. As presented in **Figure 1**, the treatment system can be conceptualized as a complex electric circuit network, where the head within tumor is equivalent to a black box, and electrode arrays on the scalp serve as the accessible nodes.

This study introduces and delves into the concept that, owing to the contrasting electrical properties of normal and malignant brain tissue (8), any alterations in tumor size and composition result in modifications to the head’s intricate black box circuit network. Real-time monitoring on the electrical impedance changes through TTFields electrodes can function as a method for detecting changes in the tumor undergoing TTFields treatment. According to the measurements, we can evaluate the effectiveness of the treatment. This approach replaces arbitrary timings for medical imaging follow-ups with follow-ups that hold clinical significance. If the impedance change abnormally, it could indicate that the treatment is ineffective, prompting a need for modification in the treatment parameters. The precise and rigid placement of electrodes, optimized for the targeted delivery of electric fields to the tumor, is likely to enhance the sensitivity of this monitoring technique to any changes in tumor dimensions, as the electrodes deliver the strongest electric fields to the tumor.

This monitoring technique has the potential to advance our fundamental understanding of the TTFields tissue ablation process and may evolve into a method for continuous assessment of treatment success throughout the procedure. It is worth noting that evaluate tissue composition change by measuring electrical impedance is not a novel concept. In fact, it forms the foundation for electrical impedance tomography (EIT) (9, 10) and magnetic induction tomography (11). Additionally, it is closely related to clinical applications, such as monitoring internal bleeding in the brain (12, 13).

In this paper, to investigate this concept, we have created an in silico finite element simulation model that simulates a TTFields treatment protocol within the brain. Through this model, we have calculated the alterations in impedance across the TTFields electrodes, considering variables such as tumor size, location, and frequency. We have then established a correlation between impedance changes and variations in the tumor’s dimensions, assuming a known tumor location relative to the electrodes. These correlations serve as a means to evaluate how sensitive these measurements are to changes in the tumor’s size. While we should emphasize that this study represents an initial theoretical exploration, the results suggest that this approach may hold clinical significance and value.

## 2 Materials and methods

**Figure 2:**
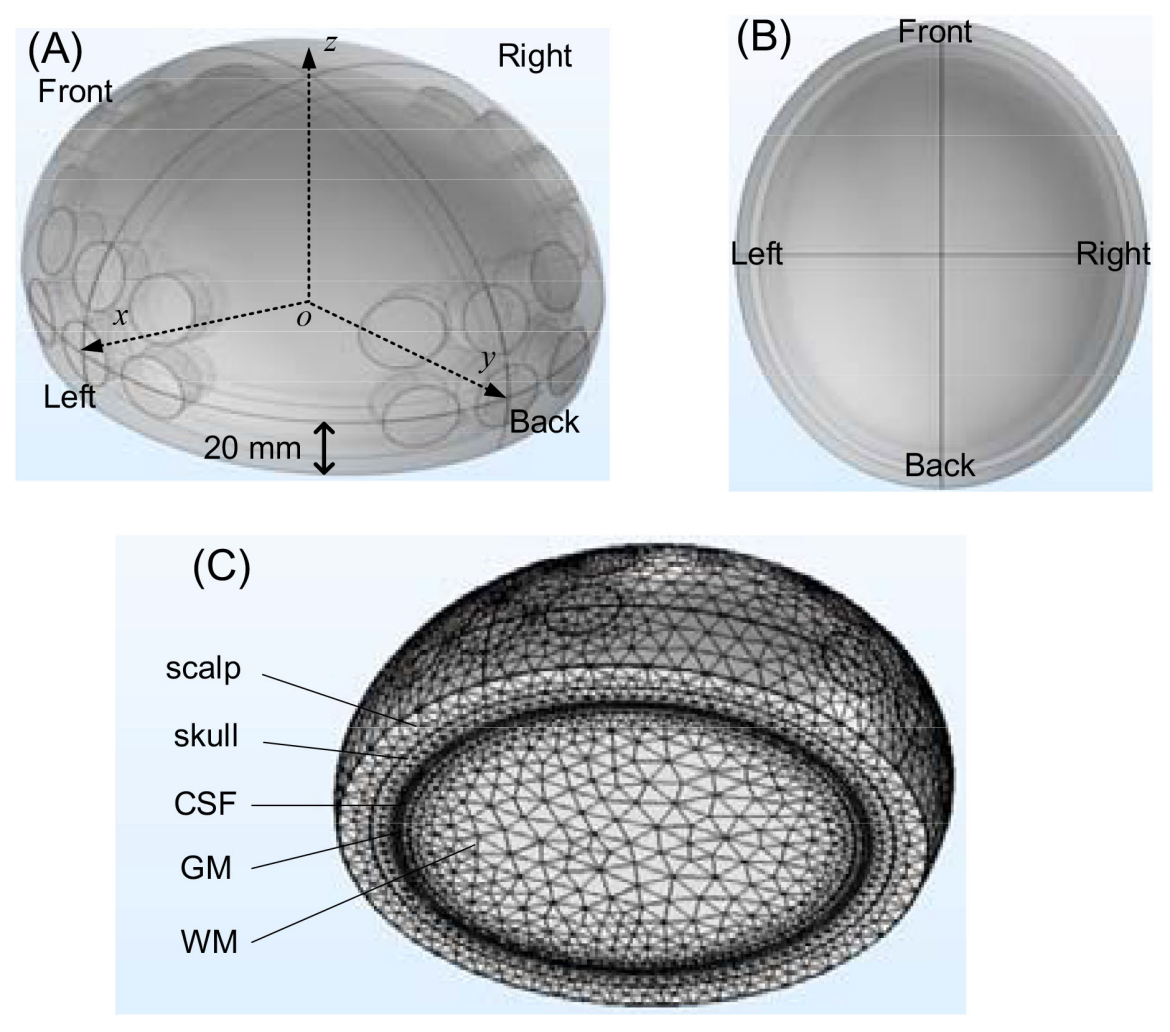
The head model built in COMSOL: **(A)** front view, **(B)** top view, **(C)** meshing result.

An in silico experimental configuration of a human head, with the brain, a tumor and the TTFields electrodes was developed in Multiphysics simulation COMSOL (version: 5.3), presented in **Figure 2A** (the front view) and **Figure 2B** (the top view). The brain was modeled by a half ellipsoid, with dimensions of 83 mm x 73 mm x 68 mm, relative to the ellipsoid centroid, in the x, y and z axis, respectively. The structure comprises five layers arranged from outer to inner layers, specifically the scalp, skull, cerebrospinal fluid (CSF), gray matter (GM), and white matter (WM). The first four layers, from the exterior to the interior are modeled as shells with a thickness of 8 mm, 6 mm, 0.75 mm, and 2 mm, in the respective order. The interior white matter fills the remainder of the half ellipsoid. These typical life-size dimensions of the head of an adult were drawn from existing publications and anatomical data (14–18). The TTFields treatment electrodes were simulated by four electrode arrays, attached to the scalp on the posterior, anterior, right and left sides of the head. Each electrode has a radius of 10 mm (19). An array six electrodes is constructed with a spacing of 5 mm between each electrode, as shown in **Figure 2A**. These TTFields treatment electrodes will serve as the sensors to detect the changes in impedance of the head, caused by changes in the tumor dimensions. The GBM tumor is shaped as a sphere and its size and location will be changed to simulate different tumor conditions. The mesh of the head model is composed of 590675 elements and 100038 nodes, as shown in **Figure 2C**.

We used the frequency domain AC/DC module in COMSOL to analyze the model. When the frequency is sub-MHz, the wavelength significantly exceeds the head’s size, hence the quasi steady approximation of electromagnetic field is applicable. However, when the frequency is above about 200 MHz, the quasi steady approximation fails and the displacement current is considered in the mathematical model. The electrical characteristics of various head components are sourced from the ITIS tissue properties database (20). Previous studies show that the tumor has an electrical conductivity and relative permittivity significantly surpassing those of the surrounding healthy tissue, ranging from several times to ten times higher (21–23). As a conservative estimate, we set the electrical properties of the tumor to be double those of surrounding white matter. Through estimation, we set a contact impedance of 1kΩ to simulate the insulation impedance of the ceramic between the electrodes and shaved scalp skin. In the simulation, the voltage applied between two opposite arrays of six electrodes is taken to be 50 V. This is a typical amplitude used to generate the desired TTFields intensity (1-2 V/cm) in the brain, as recommended in (24). The theoretical study will employ various frequencies, ranging from 5 kHz to 500 MHz, to investigate the frequency characteristic and find an optimal detection frequency.

## 3 Results and discussions

The section initially investigates the variations in impedance affected by tumor size, considering different factors: A) Frequencies, B) Locations of electrode array pairs, C) Tumor locations. Subsequently, it presents the signal-to-noise effect of the voltage source in part D).

### 3.1 Effect of frequency

**Figure 3:**
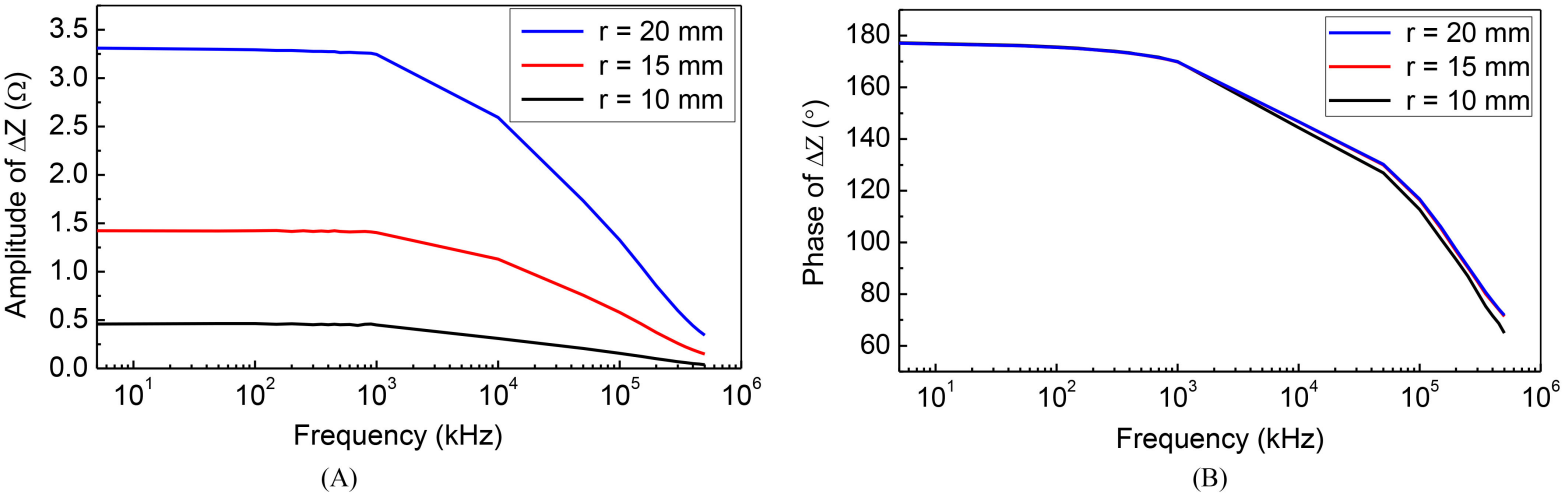
Frequency characteristic of impedance change: **(A)** amplitude-frequency **(B)** phase-frequency.

Due to the frequency-dependent character of tissues’ electrical properties (20). In this part of the study, we have placed the tumor at a specific location and calculated the impedance between the TTFields electrodes for various size tumors over a frequency spectrum spanning from 5 kHz to 500 MHz. The electric properties of the tissues were set as functions of the scanning frequency according to the data in (20). **Figure 3** was obtained for a spherical tumor located at, x = 20 mm, y = 0 mm, z = 0 mm relative to the centroid of the ellipsoid. The COMSOL simulation was performed for three radii of the tumor, r = 10 mm, 15 mm and 20 mm. The change in impedance between the left and right TTFields electrode arrays was calculated in comparison to a brain without a tumor. The change in amplitude and phase depended on the frequency, are illustrated in **Figure 3**. The curves exhibit a dispersion pattern, which is characteristic of the frequency-dependent properties inherent to biological matter (25). This is to be expected as the electrical properties of the tissues used in this model where taken from the literature. The change in amplitude due to the presence of a tumor increases with a decrease in frequency to 10^3^ kHz, after which the disparity diminishes with an elevation in frequency. In contrast, the change in impedance phase shift is minimal at lower frequencies and only becomes noticeable at higher frequencies above 100 MHz, although it still remains relatively small.

Typical TTFields frequencies are ranging from 100 kHz to 300 kHz (26), as this range has been found to yield the most significant therapeutic benefits. Interestingly, changes in tumor size coincidentally result in substantial alterations in impedance amplitude within the identical frequency range employed for treatment administration. Recording these changes in amplitude at the specific frequency of 200 kHz presents a technologically straightforward approach to monitor variations in tumor size. Importantly, such a modification can be easily incorporated into existing clinical TTFields devices. Consequently, our subsequent numerical investigations will focus on assessing the impact of various parameters on the impedance amplitude change by setting the frequency as 200 kHz. This approach aligns directly with what we consider the preferred method for evaluating the therapeutic efficacy of TTFields in brain tumor treatment.

### 3.2 Effects of electrode array pairs location

**Figure 4:**
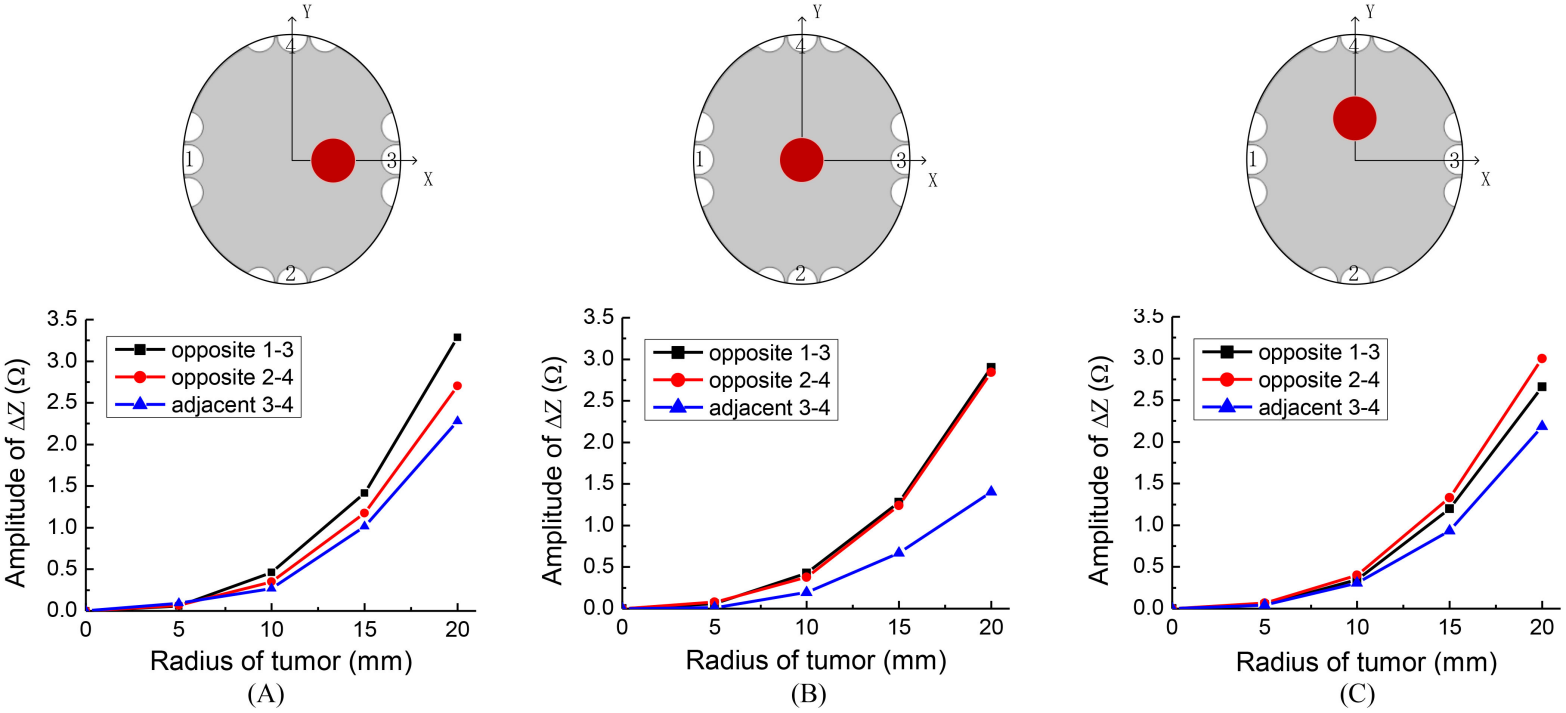
Sketches of different tumor locations and the impedance change results of different detection patterns, locations of tumor are: **(A)** (20, 0, 0), **(B)** (0, 0, 0), **(C)** (0, 20, 0).

TTFields electrode arrays are typically arranged in two opposing configurations, forming orthogonal pairs. Different impedance values can be measured by selecting opposite or adjacent electrode array pairs. For ease of reference, we assigned labels to the electrode arrays as depicted in the upper row of **Figure 4**. We defined the pairs of electrode arrays 1-3 and 2-4 as opposite detection pattern, and 3-4 as the adjacent detection pattern. In this section, our objective is to investigate the correlation between measurement sensitivity and the chosen detection pattern. In these investigations, the excitation voltage is 50 V, 200 kHz. We evaluate the impedance amplitude change between different TTFields electrode arrays pairs, as a function of tumor size at three typical locations of tumors. The tumors were placed at three different, x, y, z locations with values in mm, (20, 0, 0); (0, 0, 0) and (0, 20, 0). **Figure 4** provides insights into the alteration in impedance amplitude concerning tumor size relative to the healthy brain without a tumor, considering various array pairs.

**Figure 4** indeed illustrates that the opposite detection pattern exhibits the highest sensitivity to changes in tumor size. Given this observation, we will adopt the opposite detection pattern in the subsequent simulations. This choice aligns tentatively with the recommended detection pattern for clinical applications.

### 3.3 Effects of tumor location

In the preceding sections, we have established that the highest sensitivity for monitoring changes in tumor size during TTFields treatment is achieved by measuring the amplitude change of impedance between opposite TTFields electrode arrays pairs at a standard treatment frequency. In this section, we will explore the sensitivity of these measurements concerning the tumor’s position form the opposite detection electrode arrays.

**Figure 5:**
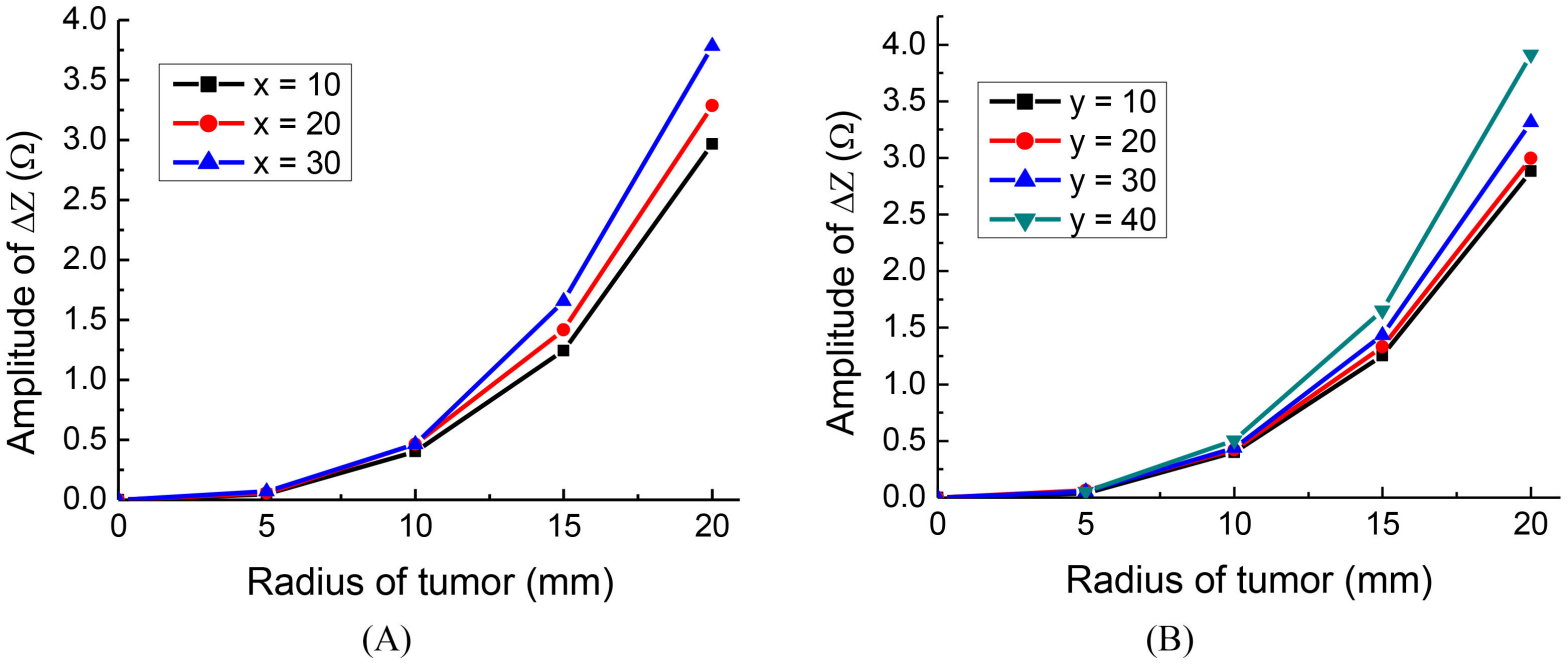
The impedance change of different tumor positions, tumor on: **(A)** x axis, **(B)** y axis.

To investigate the effect of tumor location along the x and y axes, we conducted the following simulations: For deviations along the x-axis, we positioned tumors at three different locations: x = 10 mm, 20 mm, 30 mm, with y = 0 mm and z = 0 mm. Similarly, for deviations along the y-axis, we placed tumors at four distinct locations: y = 10mm, 20 mm, 30 mm, 40 mm, with x = 0 mm and z = 0 mm. The detection electrode arrays are 1-3 for the tumor on the x-axis and 2-4 for the tumor on the y-axis. The results of the simulation are presented in **Figure 5**.

Interestingly, the findings indicate that the closer the tumor is to one of the orthogonal electrode arrays, the more substantial the change in amplitude, regardless of the tumor’s radius. This is in agreement with findings made using conventional EIT (27). The results suggest that for optimal placement, the monitoring electrode arrays should be chosen in such a way that one of the orthogonal pairs is as close as possible to the location of the tumor.

### 3.4 Signal-to-noise ratio analysis

Based on the analysis in subsection C, it is evident that when the tumor is closer to the electrodes, the impedance change is more substantial, resulting in higher monitoring sensitivity or resolution. In this subsection, as a conservative approach, we will examine the extreme condition where the tumor is located at y = 10 mm, which corresponds to the lowest monitoring sensitivity. It is important to note that the same level of noise will have a smaller impact on cases with higher monitoring resolution.

**Figure 6:**
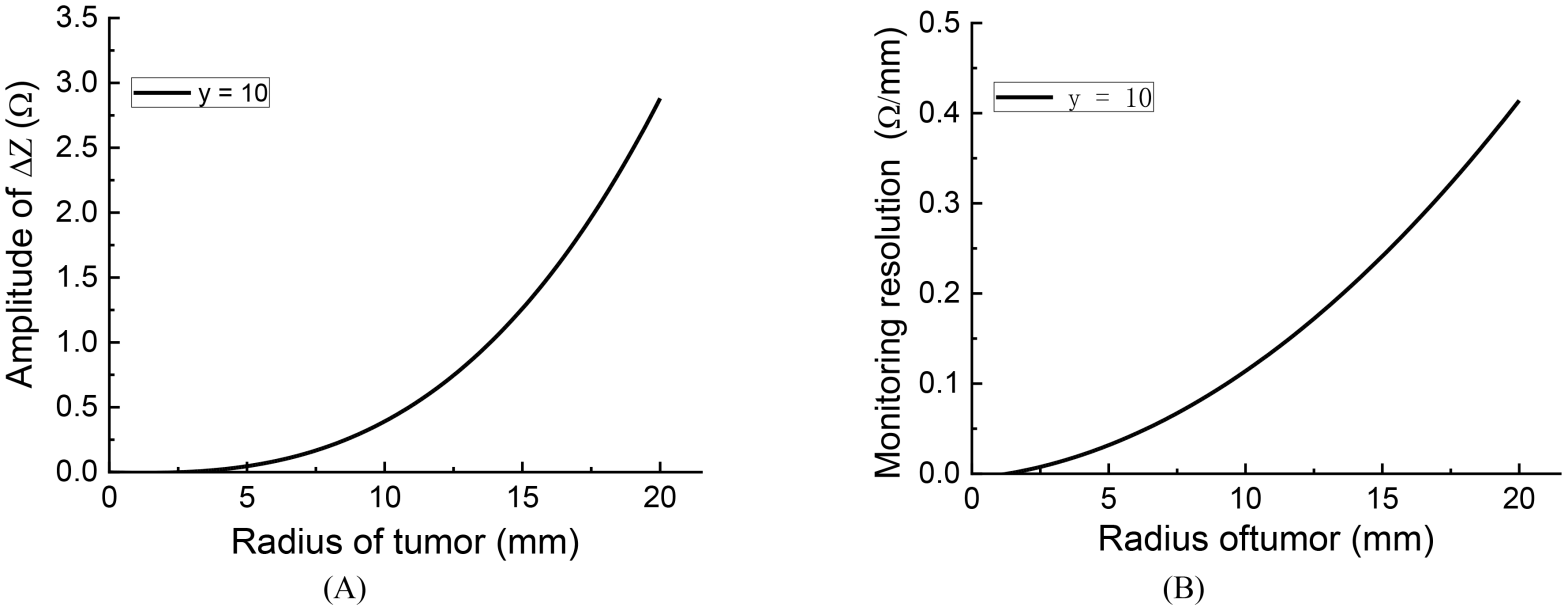
The fitting curve and monitoring resolution of impedance change curve y = 10: **(A)** fitting curve, **(B)** monitoring resolution.

To assess the monitoring resolution, we consider the first-order derivative of the impedance change concerning the tumor size. To calculate the resolution, we employed a cubic function to fit the curve for y = 10 in **Figure 5B**. The fitting function curve is depicted in **Figure 6A**, with a fitting error RMSE = 0.01452 and an Adjusted R-square = 0.9999, indicating a good fit. **Figure 6B** illustrates the monitoring resolution derived from calculating the first derivative of the fitting function. The result indicates indicate that the larger the initial tumor size, the higher the monitoring resolution.

To assess the impact of voltage source noise on the results, we introduced varying levels of noise to the source and simulated the impedance change in relation to tumor size. According to the definition of Signal-to-Noise Ratio (SNR) (28):


(1) $$\text{SNR}=20\mathrm{lg}\frac{V_{\text{s}}}{V_{\text{n}}}$$

In accordance with the formula provided, where Vs represents the accurate excitation voltage, and Vn stands for the voltage noise.

For simulating the voltage source noise, a Gaussian white noise generator was utilized in MATLAB, characterized as follows:


(2) $$V=V_{s}+awgn(V_{s},\frac{SNR}{2})$$

In the Equation 2, *V* represents the actual voltage, and the term awgn(*V*_s_, SNR/2) introduces Gaussian noise with a specific SNR to the accurate voltage *V*_s_. It is important to note that the SNR definition used in the awgn function is based on power; hence, the division by 2 is necessary to convert from voltage SNR to power SNR.

**Figure 7:**
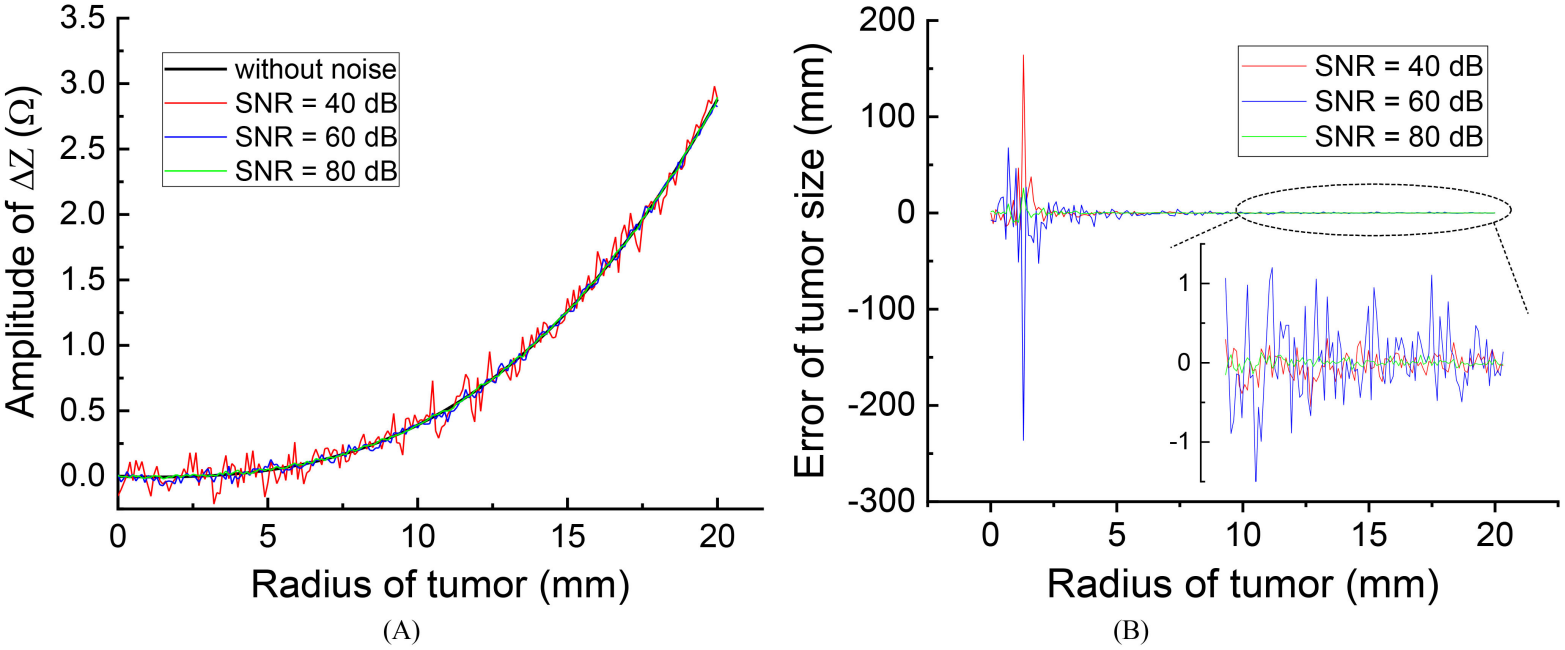
The effect of different source noise on the evaluation results: **(A)** effect on the impedance change curves **(B)** evaluation error of tumor size.

After introducing noise to the voltage source, the impedance change curves for different SNR levels, along with the assessment of errors induced by the noise, are presented in **Figure 7A**.

**Figure 7B** illustrates that the presence of noise introduces errors in the measured impedance results, with smaller errors observed at higher SNR levels. Specifically, when the initial tumor size is smaller than 5 mm, noise can lead to significant errors in tumor size evaluations. However, for initial tumors larger than 10 mm, the high monitoring resolution within this range (r > 10 mm) allows for acceptable error levels, even with an SNR as low as 40 dB, resulting in a maximum tumor size evaluation error of approximately 1.0 mm. This level of error is generally considered acceptable and can be disregarded.

In practical applications, achieving an SNR of 40 dB is feasible and not particularly challenging in hardware systems. Therefore, the anti-noise capability is sufficiently robust for monitoring tumor size by measuring impedance changes across the treatment electrodes. It is important to emphasize that for tumors smaller than 5 mm, the monitoring resolution and anti-noise capacity are reduced, making it advisable to employ more precise monitoring techniques such as MRI or CT to evaluate changes in tumor size.

## 4 Conclusions

TTFields represent a relatively new cancer therapy technology designed to combat cancer by disrupting the mitosis of cancer cells. This treatment period typically lasts several months and even years, posing a challenge for monitoring its effectiveness over time. In this study, we explored the hypothesis that real-time monitoring of tumor condition change can be achieved by measuring the impedance change through TTFields treatment electrodes. An in silico study has provided initial evidence supporting the potential value of this proposed method. Preliminary findings suggest that it is feasible to detect tumor size change by measuring amplitude change of impedance across opposite TTFields electrode pairs, utilizing typical TTFields treatment excitation (50V, 200 kHz). Implementing this technique can be straightforward, involving enhancements to the impedance measurement functionality within the existing TTFields treatment hardware system. The scalp electrode arrays will serve dual functions, delivering TTFields and serving as impedance sensors. It is crucial to acknowledge that this study represents a preliminary feasibility investigation, and further validation through clinical studies is essential. If proven successful, this monitoring system could emerge as a valuable augmentation to TTFields cancer treatment technology, offering a means to monitor treatment effectiveness in real-time, potentially enhancing patient outcomes and care.

## Data Availability

The original contributions presented in the study are included in the article/supplementary material. Further inquiries can be directed to the corresponding authors.
